# Hepatic Amyloid Beta-42-Metabolizing Proteins in Liver Steatosis and Metabolic Dysfunction-Associated Steatohepatitis

**DOI:** 10.3390/ijms25168768

**Published:** 2024-08-12

**Authors:** Simon Gross, Lusine Danielyan, Christa Buechler, Marion Kubitza, Kathrin Klein, Matthias Schwab, Michael Melter, Thomas S. Weiss

**Affiliations:** 1 Children’s University Hospital (KUNO), University Hospital Regensburg, 93053 Regensburg, Germany; 2 Department of Clinical Pharmacology, University Hospital Tuebingen, 72076 Tuebingen, Germany; 3 Department of Internal Medicine I, University Hospital Regensburg, 93053 Regensburg, Germany; 4 Dr. Margarete Fischer-Bosch Institute of Clinical Pharmacology, Stuttgart and University of Tuebingen, 72076 Tuebingen, Germany; 5 Department of Biochemistry and Pharmacy, University Tuebingen, 72076 Tuebingen, Germany; 6 Center for Liver Cell Research, University Hospital Regensburg, 93053 Regensburg, Germany

**Keywords:** MASLD, NAFLD, MASH, NASH, steatosis, fibrosis, oxidative stress, fatty acids, amyloid

## Abstract

Amyloid beta (Aβ) plays a major role in the pathogenesis of Alzheimer’s disease and, more recently, has been shown to protect against liver fibrosis. Therefore, we studied Aβ-42 levels and the expression of genes involved in the generation, degradation, and transport of Aβ proteins in liver samples from patients at different stages of metabolic dysfunction-associated liver disease (MASLD) and under steatotic conditions in vitro/in vivo. Amyloid precursor protein (APP), key Aβ-metabolizing proteins, and Aβ-42 were analyzed using RT-PCR, Western blotting, Luminex analysis in steatotic in vitro and fatty liver mouse models, and TaqMan qRT-PCR analysis in hepatic samples from patients with MASLD. Hepatocytes loaded with palmitic acid induced APP, presenilin, and neprilysin (NEP) expression, which was reversed by oleic acid. Increased APP and NEP, decreased BACE1, and unchanged Aβ-42 protein levels were found in the steatotic mouse liver compared to the normal liver. Aβ-42 concentrations were low in MASLD samples of patients with moderate to severe fibrosis compared to the livers of patients with mild or no MASLD. Consistent with the reduced Aβ-42 levels, the mRNA expression of proteins involved in APP degradation (ADAM9/10/17, BACE2) and Aβ-42 cleavage (MMP2/7/9, ACE) was increased. In the steatotic liver, the expression of APP- and Aβ-metabolizing proteins is increased, most likely related to oxidative stress, but does not affect hepatic Aβ-42 levels. Consistent with our previous findings, low Aβ-42 levels in patients with liver fibrosis appear to be caused by the reduced production and enhanced non-amyloidogenic processing of APP.

## 1. Introduction

Alzheimer’s disease (AD) is a degenerative brain disorder and is the most common type of dementia. The most prevalent form of AD, which occurs late in life, is associated with the deposition of amyloid beta (Aβ) in the brain following its accumulation in the brain. The balance between Aβ protein production and clearance determines the steady-state level of Aβ, while disturbances in Aβ homeostasis lead to Aβ deposits in the brain [1].

Aβ peptides are derived from the amyloid precursor protein (APP), a transmembrane protein, through the sequential cleavage of APP by membrane-bound β-secretases and γ-secretases. This route of APP processing is termed the amyloidogenic pathway and leads to different species of Aβ, of which Aβ-42 has a deleterious effect on the aggregation and formation of plaques [1]. Therefore, APP is cleaved by β-secretase BACE1 (β-site APP cleaving enzyme 1), a membrane-bound aspartic protease, into N-terminal soluble APPsβ and a membrane-tethered βCTF (β C-terminal fragment). The latter is subjected to further processing by a γ-secretase enzyme complex containing presenilin (PSEN 1/2, PS1/2), resulting in the intracellular fragment AICD (APP intracellular domain) and Aβ [2]. In particular, PS1/2 cleaves the βCTF fragment at positions 40 and 42, yielding the protein fragments Aβ40/42 [2]. Furthermore, APP can be cleaved within the Aβ domain by membrane-bound α-secretases such as ADAMs (a disintegrin and metalloproteases), generating the soluble N-terminal fragment APPsα and the membrane-tethered αCTF (α C-terminal fragment). In this non-amyloidogenic pathway, αCTF is further processed by the γ-secretase complex into extracellular peptide p3 and the intracellular fragment AICD [2]. The Aβ protein concentration in a given compartment is not only balanced by the activation of amyloidogenic or non-amyloidogenic pathways, but also by the degradation or elimination of Aβ protein [3]. The degradation of Aβ in the brain and peripheral organs, including the liver, is performed by a variety of proteases belonging to the metalloprotease (e.g., NEP, neprilysin, ECE 1/2, endothelin-converting enzyme 1/2, ACE, angiotensin I-converting enzyme, MMP, and matrix metallopeptidase), serine protease (e.g., APEH, acylaminacyl-peptide hydrolase, MBP, myelin basic protein, IDE, and insulin-degrading enzyme), cysteine protease (Cathepsin B), and aspartyl protease (e.g., BACE2) families. Aβ-degrading proteases cooperate with other catabolic processes for Aβ elimination and can be induced or repressed by Aβ peptides itself [4].

The liver plays a central role in the human body; therefore, disturbances in hepatic functionality affect various organs, including the brain, emphasizing the significance of the liver–brain axis [3]. In this sense, the recently reported association between postoperative cognitive dysfunction after orthotopic liver transplantation and increased serum markers of dementia, including Aβ-42 [5], may reflect the importance of the hepatic clearance of these factors for intact brain function. An epidemiological study showed that comorbidities, such as liver cirrhosis, were significantly associated with mild cognitive impairment and dementia [6]. In addition, cirrhotic livers do not properly eliminate Aβ-42 from the serum [7], and serum levels of Aβ-42 are increased in patients with cirrhosis [8]. Liver tissues from patients with cirrhosis revealed decreased Aβ-42 levels and a low expression of key proteins that are known to generate and degrade Aβ-42 [9]. Furthermore, Buniatian et al. reported an unexpected role of Aβ-42 in the liver as a regulator of cell–cell interactions and the protection against liver fibrosis/cirrhosis [9,10].

Non-alcoholic fatty liver disease (NAFLD), recently noted as metabolic dysfunction-associated steatotic liver disease (MASLD), is the leading cause of chronic liver disease worldwide [11]. NAFLD/MASLD encompasses various disorders characterized by slow progression, initially caused by abnormal or excessive accumulation of lipid droplets in hepatocytes (steatosis, fatty liver), leading to metabolic dysfunction-associated steatohepatitis (MASH/NASH) and its progression towards hepatic cirrhosis, as well as an increased risk of developing liver cancer [11]. The critical determinants of disease progression are reactive oxygen species (ROS), endoplasmic stress, mitochondrial dysfunction, and lipid toxicity, leading to cell death, inflammation, and fibrogenesis [11]. Besides its impact on liver function, MASLD is also associated with a lower cognitive performance and an altered memory [12].

Despite the notion that MASLD is associated with and contributes to AD [6,13], the mechanism underlying this interplay is unclear. Given the puzzling gaps in understanding the role of hepatic Aβ clearance in different liver disorders, we sought to focus on the current work on MASLD-associated changes in the hepatic utilization of Aβ. Therefore, we analyzed hepatic Aβ levels and the gene expression of proteins involved in the generation, degradation, and transport of Aβ proteins in liver tissue samples from patients with different stages of MASLD. Furthermore, we assessed APP- and Aβ-metabolizing proteins in steatotic mouse livers and hepatocytes upon lipotoxic stress induced by saturated fatty acids.

## 2. Results

### 2.1. Saturated Palmitic Acid Induces the Gene Expression of APP and Its Metabolizing Proteins In Vitro, Which Is Attenuated by Mono-Unsaturated Oleic Acid

**Figure 1:**
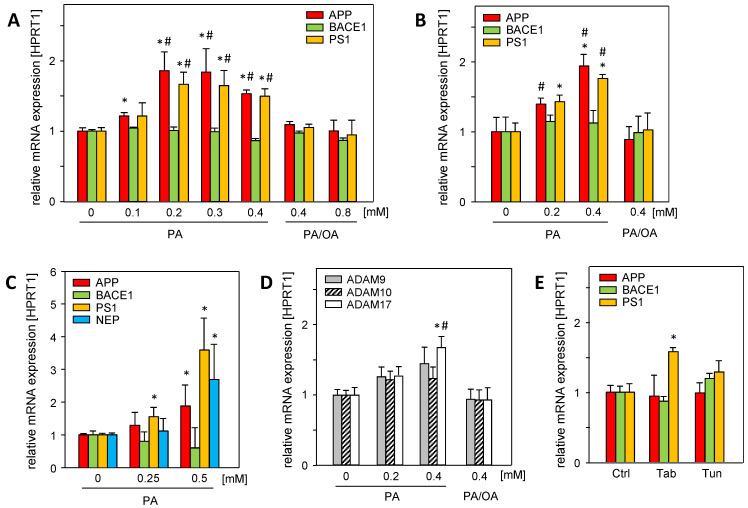
Palmitic acid (PA) induces the mRNA expression of APP and its metabolizing proteins in vitro, which is reduced by mono-unsaturated oleic acid (OA). (**A**) Huh7 and (**B**) HepG2 cells were treated without or with indicated concentrations of PA or PA/OA (1/2) for 24 h, and (**C**) primary human hepatocytes (PHHs) were treated without or with PA for 24 h. (**D**) Huh7 cells were treated without or with PA or PA/OA (1/2) for 24 h. (**E**) HepG2 cells were treated with endoplasmic reticulum (ER) stress inducers thapsigargin (Tab, 0.5 µM for 6 h) or tunicamycin (Tun, 10 µg/mL for 16 h). The mRNA levels of genes involved in the amyloidogenic (APP, BACE1, PS1, and NEP) and non-amyloidogenic (ADAM9, ADAM10, and ADAM17) pathways of APP and its metabolizing genes were analyzed using qRT-PCR, and were normalized to HPRT1 (three independent experiments, mean ± SEM). * *p* < 0.05 differs from untreated control (0, Ctrl); # *p* < 0.05 differs from 0.4 mM or 0.8 mM PA/OA treatment.

Hepatic steatosis, a major hallmark of MASLD, is caused by the disruption of lipid homeostasis and is associated with fatty acid-mediated cell stress, which can be analyzed using in vitro models of steatosis [14]. Saturated palmitic acid (PA) significantly induced the mRNA expression of amyloid precursor protein (APP), γ-secretase presenilin (PS1), and α-secretase ADAM17 in Huh7 and HepG2 cells (Figure 1A,B). Neprilysin mRNA (NEP/MME), the main Aβ-42 degrading enzyme, was barely expressed in hepatoma cell lines. In primary human hepatocytes, PA treatment induced the mRNA expression of APP, PS1, and neprilysin (NEP) (Figure 1C), whereas β-secretase BACE1 was not affected.

The treatment of the cells with a mixture of saturated PA and mono-unsaturated oleic acid (PA/OA, ratio 1/2) abolished the enhanced expression (Figure 1A,B,D) of APP- and Aβ-42-generating genes. Since oleic acid attenuates the deleterious effects of saturated fatty acids, such as PA [15], it is likely that cellular oxidative stress caused by PA is responsible for enhanced mRNA expression. In addition, PA treatment induces endoplasmic reticulum (ER) stress [16]. The ER stress inducers tunicamycin (Tun) and thapsigargin (Tab) had no effect on APP and BACE1 mRNA levels, while PS1 mRNA expression was only increased by Tab (Figure 1E). This largely excludes PA-induced ER stress as a cause of the enhanced expression of these genes.

### 2.2. Hepatic Expression of APP and Its Metabolizing Protein, but Not Aβ-42, Levels Are Altered in a Fatty Liver Mouse Model

**Figure 2:**
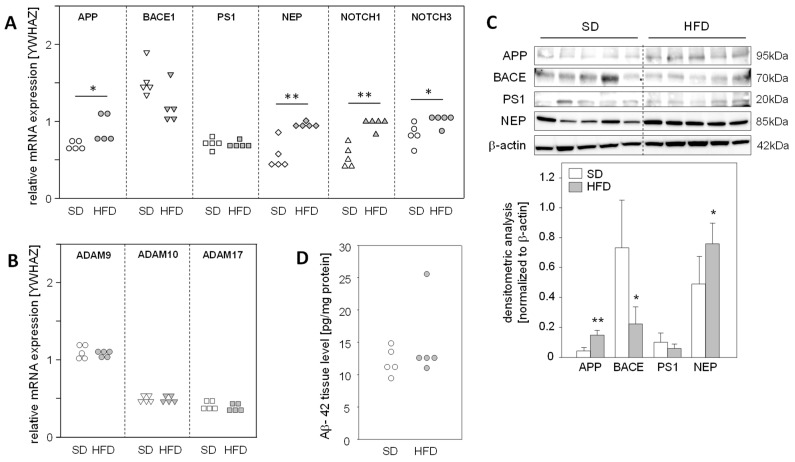
Expression of APP, its metabolizing proteins, and the hepatic levels of Aβ-42 in mice fed a high-fat diet. Male mice were fed a standard diet (SD) or a high-fat diet (HFD) for 14 weeks, resulting in hepatic steatosis in the HFD group. Hepatic liver tissue was analyzed for mRNA expression of (**A**) APP- and Aβ-42-generating genes, as well as γ-secretase (PS1) substrates NOTCH1/3 and (**B**) non-amyloidogenic pathway-related genes. The mRNA levels were analyzed using qRT-PCR and were normalized to YWHAZ (n = 5). (**C**) Total protein extracts were isolated from liver samples and Western blot analysis using specific antibodies was performed with β-actin as loading control. Relative protein abundance was determined using densitometric analysis and was normalized to the loading control. (**D**) Hepatic Aβ-42 levels in samples from mice fed a standard diet (SD) or a high-fat diet (HFD). Aβ-42 concentrations were determined using multiplex analysis in homogenates from liver tissue. Data are presented either as data points or mean ± SD (n = 5); * *p* < 0.05 and ** *p* < 0.01.

The fact that a high-fat diet (HFD) for 14 weeks leads to the development of fatty liver with no signs of fibrosis in mice [17] led us to use this model to explore the impact of hepatic steatosis on the expression of APP, APP degradation, and Aβ-42-producing proteins in the liver. We found a significantly enhanced mRNA expression of APP, NEP, and NOTCH1/3, substrates of γ-secretase PS1, in steatotic livers of HFD mice (Figure 2A). Protein analysis demonstrated increased APP and NEP levels in the livers of HFD-fed mice, whereas BACE1 was reduced in comparison to the normal livers of mice fed a standard diet (SD) (Figure 2C). Interestingly, mRNA expression of the α-secretases of the non-amyloidogenic pathway, ADAM9/10/17 (Figure 2B), and hepatic Aβ-42 levels were unaltered in HFD mice compared to standard diet (SD) mice (Figure 2D). These findings support the assumption that in vivo hepatic fat accumulation, and consequently increased oxidative stress, results in the enhanced expression of APP and altered levels of Aβ-42-generating proteins, but these changes in protein expression do not affect hepatic Aβ-42 levels.

### 2.3. Hepatic Aβ-42 Levels Are Reduced in Patients with MASH

**Figure 3:**
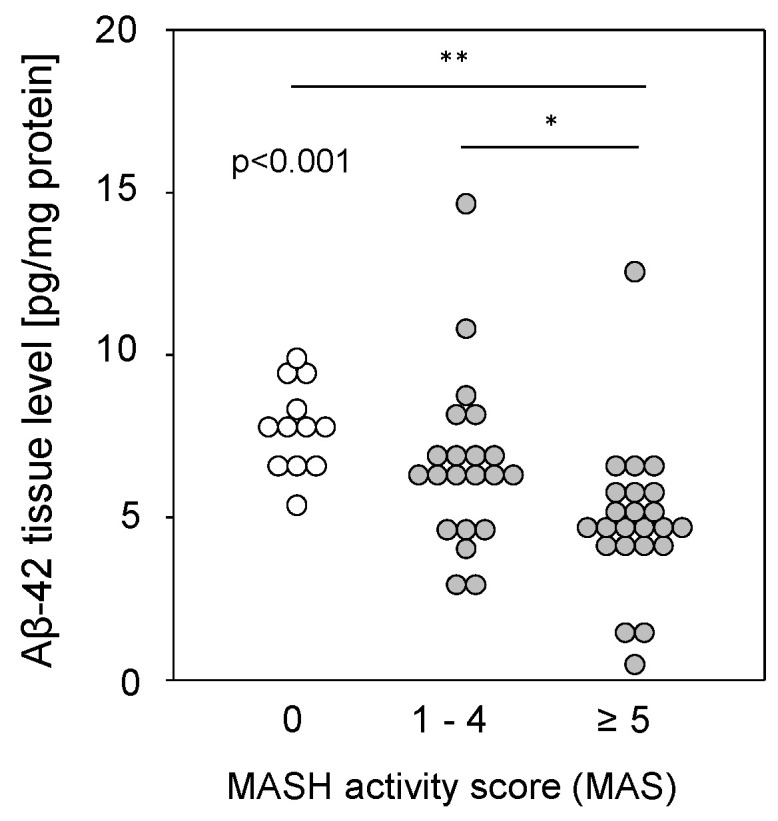
Hepatic Aβ-42 levels in samples from patients with MASLD. Aβ-42 concentrations were determined using multiplex analysis in tissue homogenates from patients with MASH (MAS ≥ 5; n = 23; 4.91 ± 2.30), steatosis (MAS 1–4; n = 21; 6.67 ± 2.63), or control liver (MAS 0; n = 12; 7.78 ± 1.35). * *p* < 0.05 and ** *p* < 0.01 were considered as significantly different.

Liver tissue samples from patients with MASLD (see Table S2), from patients with a metabolic-dysfunction associated steatohepatitis (MASH) activity score (MAS) of 1–4 (steatosis) or 5–8 (MASH), and from patients with histopathologically proven non-diseased livers (MAS = 0) were homogenized and assessed for Aβ-42 using multiplex analysis. Aβ-42 tissue levels were found to be significantly reduced in the livers of patients with MASH compared to those with simple steatosis or controls (Figure 3). The reduction was independent of type 2 diabetes (T2D), BMI, hypertension, hypercholesterolemia, or the sex of the patients. It was previously shown that reduced Aβ-42 levels were found in liver tissue from patients with fibrosis or cirrhosis [9], but a comparison of Aβ-42 levels with histological fibrosis scores in samples with MAS ≥1 did not show a significant difference between no, mild to moderate, or severe fibrosis and cirrhosis (Figure S1A). Furthermore, Aβ-42 levels in steatotic samples without fibrosis did not differ according to tissue steatosis grade (Figure S1B). Therefore, regarding MASH, the observed diminished Aβ-42 liver tissue levels may correspond to a multifactorial disease (hepatic steatosis combined with fibrosis) rather than to a single pathological condition.

### 2.4. mRNA Expression of APP, APP Degradation, and Aβ-42-Processing Proteins Is Altered in Liver Tissue from Patients with MASH

**Figure 4:**
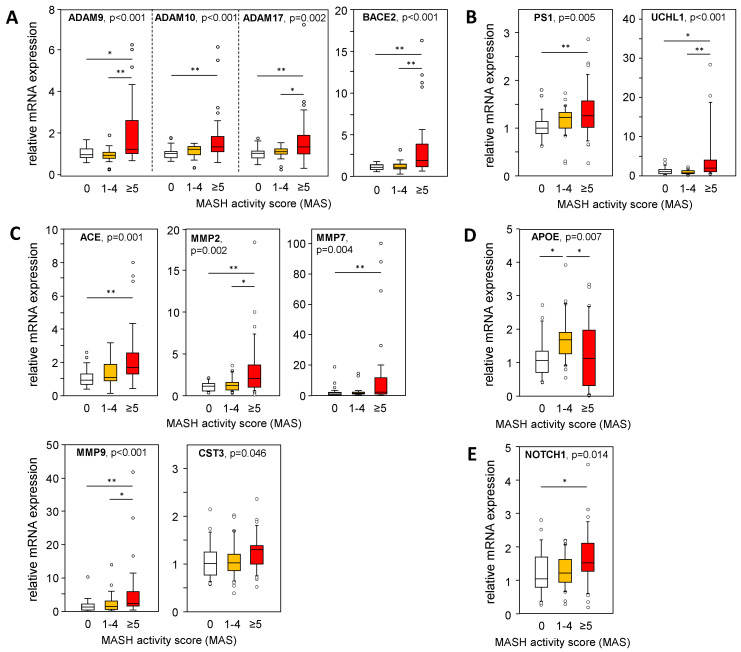
Expression of APP- and Aß-42-processing proteins in liver samples from patients with MASLD. The mRNA expression of proteins involved in the processing of APP and Aβ-42 via (**A**) non-amyloidogenic and (**B**–**D**) amyloidogenic pathways was analyzed: (**A**) degradation of APP, (**B**) degradation of APP followed by processing towards Aβ-42 formation, (**C**) degradation of Aβ-42, (**D**) binding / transport of Aβ-42, and (**E**) alternative substrate of γ-secretase PS1. mRNA expression was analyzed in hepatic tissue samples from patients with MASH (MAS ≥ 5, n = 36), hepatic steatosis (MAS 1–4, n = 30), and normal liver tissue (MAS 0, n = 26) using qRT-PCR followed by normalization to three housekeeping genes—GUSB, HPRT1, and TBP (see Table S3). Data are presented as box blots displaying median values, lower and upper quartiles, and the range of the values (whiskers), with outliers shown as circles (values between 1.5 and 3 times the interquartile range). Statistical differences were analyzed using the Kruskal–Wallis test with post hoc Bonferroni correction. * *p* < 0.05, ** *p* < 0.01.

**Figure 5:**
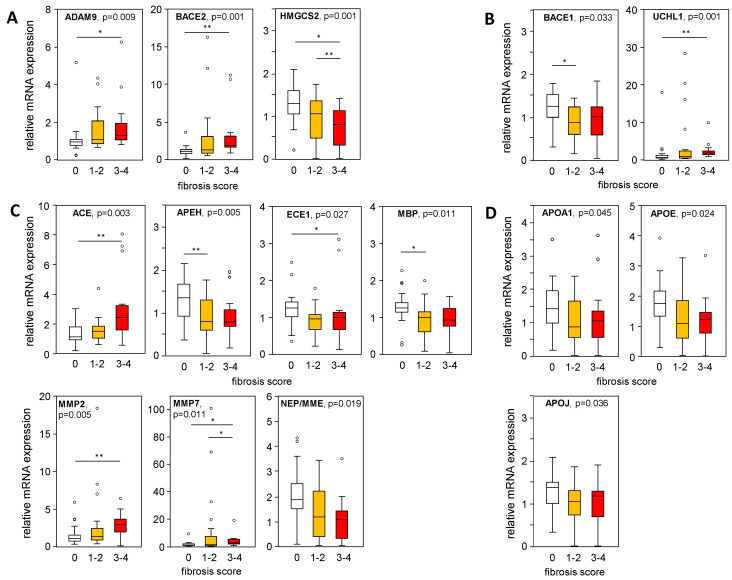
Expression of APP-processing proteins in relation to liver tissue fibrosis scores. mRNA expression of proteins processing APP and Aβ-42 were plotted regarding their histologically proven fibrosis grade in liver samples from patients with an MAS ≥ 1 (steatosis and MASH). (**A**) Non-amyloidogenic pathway and (**B**–**D**) amyloidogenic pathway of APP and Aβ-42 processing: (**A**) degradation of APP, (**B**) degradation of APP followed by processing towards Aβ-42 formation, (**C**) degradation of Aβ-42 and (**D**) binding/transport of Aβ-42. mRNA expression was analyzed using qRT-PCR, followed by normalization to three housekeeping genes—GUSB, HPRT1, and TBP (see Table S3). Data are presented as box blots displaying median values, lower and upper quartiles, and the range of the values (whiskers), with outliers shown as circles (values between 1.5 and 3 times the interquartile range). Fibrosis score: 0, n = 27; 1–2, n = 23; 3–4, n = 14. Statistical differences were analyzed using the Kruskal–Wallis test with post hoc Bonferroni correction. * *p* < 0.05, ** *p* < 0.01.

**Figure 6:**
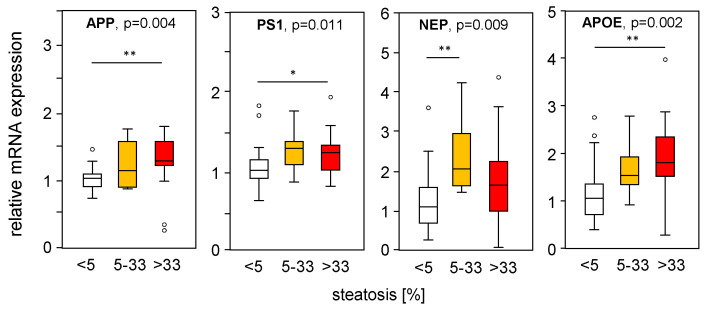
Expression of APP-processing proteins in relation to liver tissue steatosis grade. mRNA expression of genes processing APP and Aβ-42 were plotted according to the histologically proven steatosis grade in liver samples from patients without hepatic fibrosis. mRNA expression was analyzed using qRT-PCR, followed by normalization to three housekeeping genes—GUSB, HPRT1, and TBP (see Table S3). Data are presented as box blots displaying median values, lower and upper quartiles, and the range of the values (whiskers), with outliers shown as circles (values between 1.5 and 3 times the interquartile range). Steatosis grade: 0 < 5, n = 26; 5–33, n = 10; >33, n = 17. Statistical differences were analyzed using the Kruskal–Wallis test with post hoc Bonferroni correction. * *p* < 0.05, ** *p* < 0.01.

A larger cohort of liver samples from patients with MASLD (see Table S1) was analyzed for the mRNA expression of genes involved in APP and Aß-42 processing via non-amyloidogenic and amyloidogenic pathways. We used a TaqMan RT-PCR-based high-throughput platform to analyze a variety of genes involved in APP and Aβ-42 metabolism (Table S3) in liver samples from patients with an MAS of 1–4 (steatosis) or 5–8 (MASH), and from patients with histopathologically proven non-diseased livers (MAS = 0). In Figure 4, Figure 5 and Figure 6, only data with a significant difference in the mRNA expression of the analyzed genes are shown. In MASH samples, we found a significantly increased mRNA expression of members of the α-secretase family, ADAM9/10/17, and BACE2 (Figure 4A), all known for their APP cleavage activities, thereby fueling the non-amyloidogenic pathway and implicating lower Aβ-42 tissue levels. In addition, UCHL1 expression, a negative regulator of BACE1 activity that is mostly responsible for Aβ-42 production, was enhanced in MASH samples, indicating diminished Aβ-42 levels (Figure 4B). Furthermore, γ-secretase PS1/PSEN1 (Figure 4B) and their alternative cleavage substrate NOTCH 1 (Figure 4E) was increased in MASH. Aβ-42 can be degraded by several enzymes, of which ACE and the matrix metalloproteinases MMP2, MMP7, and MMP9 are significantly elevated in MASH (Figure 4C). These findings are further supported by an inverse correlation between Aβ-42 levels and the mRNA expression of ADAM9/10/17, UCHL1, ACE, MMP2, and MMP9 in the MASH samples (Table S6). Overall, low hepatic Aβ-42 levels in patients with MASH appear to be dependent on activated non-amyloidogenic and hindered amyloidogenic pathways.

### 2.5. Fibrotic Rather Than Steatotic Liver Tissue Conditions Are Responsible for Differentially Expressed APP- and Aβ-42-Processing Genes

Furthermore, mRNA expression levels of APP-processing genes were plotted in liver samples from patients with an MAS ≥ 1, indicating steatosis or MASH, stratified according to their histological fibrosis score. In liver samples with mild/moderate to severe fibrosis compared to the absence of fibrosis, we found a significantly increased expression of ADAM9, BACE2, and UCHL1, as well as a reduced BACE1 expression (Figure 5A,B), corresponding to APP processing towards a non-amyloidogenic pathway. In fibrotic MASLD samples, the Aβ-42-degrading enzymes ACE, MMP2, and MMP7 were enhanced, while the expression of APEH, ECE1, and MBP was reduced (Figure 5C). APOA1, APOE, and APOJ expression, all responsible for Aβ-42 binding and transport, declined with higher fibrosis scores (Figure 5D). Furthermore, correlation analysis of mRNA expression levels with histological grades of inflammation (Table S7) showed only weak correlations for ADAM9, HMGCS2, MMP2, MMP7, NEP, APOE, and NOTCH3.

To assess the impact of hepatic steatosis on APP-processing gene expression, we divided the mRNA expression of liver samples from MASLD patients according to their histological steatosis grade, thereby excluding samples with fibrosis. Interestingly, we found increased APP, PS1, and NEP in steatotic liver tissue compared to normal liver tissue (Figure 6), which is in line with the PA-induced mRNA expression found in vitro (Figure 1A–C) and in part in HFD mice (Figure 2A–B). APOE expression was induced under steatotic conditions and was reduced under fibrotic conditions (Figure 4D). APOE expression was positively correlated with steatosis grade (Figure 6), reduced in samples with an MAS ≥ 5 compared to MAS 1–4 (Figure 4D), and negatively correlated with fibrosis grade (Figure 5D). The mRNA expression of the studied genes did not change with underlying T2D, BMI, hypertension, hypercholesterinemia, or sex of the patients.

## 3. Discussion

MASLD, the leading cause of chronic liver disease, is associated with cognitive impairment and AD [13], as well as dyslipidemia, which correlates with mental status in patients with AD [12]. Additionally, diet-induced MASLD triggers the pathological signs of AD in wild-type mice. This effect was enhanced in APP- [18] and APP/PS1 [19]-transgenic mice. A recent report showing neurodegeneration in juvenile pigs with diet-induced MASLD provided evidence of gut probiotics for Aβ levels and memory loss [20]. Hypercholesterolemia may also be related to an increased risk of developing AD or mild cognitive impairment in humans [21]. In addition, oxidative stress and its related products are recognized as being involved in the onset of AD [22]. These findings strongly confirmed the liver–brain axis, with the liver as the central organ of lipid metabolism. While most reports have addressed the consequences of disturbed lipid metabolism on brain physiology and disease development, little is known about its impact on hepatic Aβ metabolism.

Since impaired peripheral clearance of Aβ by the liver is a feature of MASLD [3], and due to the fact that free fatty acids have been shown to be responsible for oxidative and endoplasmic reticulum stress in hepatocytes [15,16], we analyzed the impact of free fatty acids on the hepatic Aβ levels and mRNA expression of its metabolizing proteins. The treatment of hepatocytes with saturated PA enhanced the mRNA expression of APP, PS1, NEP, and ADAM17, and this effect was abrogated by monounsaturated oleic acid. This suggests that the hepatic accumulation of fatty acids [14,23] is not the main cause of Aβ gene regulation, which seems to be induced by increased oxidative stress upon PA exposure. Palmitic acid has been shown to increase reactive oxygen species (ROS) production by promoting the uncoupling of glycolysis and tricarboxylic acid (TCA) cycle fluxes, which enhances redox imbalance and the formation of reactive oxygen intermediates, leading to ER stress and apoptosis [24]. In our in vitro studies, ER stress may play a minor role in the regulation of the gene expression of Aβ-metabolizing proteins, since ER stress inducers showed no effect, except increased PS1 expression after tapsigargin treatment. We have previously reported that PA treatment induces oxidative stress and subsequently activates different pathways, leading to NF-κB activation [23]. Furthermore, oxidative stress has been shown to induce ADAM17 expression in platelets via p38 MAPK signaling [25], which parallels our findings of PA-induced ADAM17 expression and its abolishment by OA addition (less p38 activation) in hepatoma cells. However, there are conflicting results regarding Aβ-metabolizing gene expression related to oxidative stress, reporting that PA upregulates BACE1 expression in skeletal muscle cells [26]. In contrast, in vitro studies in human neural cells demonstrated no effect on BACE1 transcription by EGF (activating Erk1/2 and p38), PMA (phorbol 12-myristate 13-acetate) (activating PKC), or IL-1β and TNF (activating NFkB) [27]. We found that the addition of OA abolishes the PA-induced expression of Aβ-metabolizing proteins, which points to a role of oxidative stress in Aβ metabolism, as shown recently in neural cells [28].

In fatty liver tissue from mice fed an HFD [17], we found an enhanced hepatic APP and NEP expression compared to normal liver tissue. These findings are consistent with previous reports showing increased hepatic APP expression in leptin-deficient mice (ob/ob mice) with advanced liver steatosis and oxidative stress [29]. Furthermore, in patients with obesity, diabetes, and MASLD, a high NEP expression has been found in the liver along with an enhanced NEP activity in the blood, with hepatic fat accumulation being a driver of increased circulating NEP [30]. Additionally, we found unchanged BACE1 mRNA in vitro, but a reduced BACE1 protein expression in the livers of HFD mice, which might be due to increased Nrf2 (nuclear factor erythroid-derived 2-related factor 2, NFE2L2) activity under oxidative stress in hepatic steatosis [24]. Nrf2, a transcription factor that induces a variety of cytoprotective and detoxification genes, negatively regulates BACE1 expression independently of redox regulation in humans and mouse models of AD [31]. In addition, Nrf2 expression levels were upregulated in the livers of patients with MASLD and diet-induced obese mice [32]. The expression of α-secretases ADAM9, ADAM10, and ADAM17, and notably Aβ-42 levels, were not changed in steatotic compared to normal liver tissue in HFD mice. A limitation of the mouse model in this study is that male mice were used and the inclusion of female subjects should be considered for future research. In conclusion, APP- and Aβ-42-metabolizing proteins may be regulated by fatty liver conditions, but do not affect hepatic Aβ-42 levels.

To our knowledge, our study is the first attempt to characterize the expression of APP, Aβ-42, and their metabolizing genes in the liver tissue of patients with MASLD. Here, we show reduced Aβ-42 levels in tissue samples from patients with a moderate to severe MAS compared to those from patients with a low MAS (steatosis) or a normal liver. Even though this decline in Aβ-42 levels in liver tissue does not correlate with corresponding histopathological fibrosis scores, it may be associated with fibrogenesis in MASLD, according to our previous report on lower hepatic Aβ-42 concentrations in cirrhosis [9]. The Aβ protein is generated from APP via the amyloidogenic pathway, and hepatic Aβ-42 levels are determined by the rate of generation and degradation. APP is an integral membrane protein and a precursor protein of Aβ-42 that plays a central role in AD pathogenesis [33]. Bioinformatics studies have revealed that APP expression is increased in severe versus mild MASLD livers and might be involved in the development of MASLD [34]. We did not find an altered APP expression in hepatic samples from patients with regard to their MASH activity, but detected increased APP expression in steatotic livers from patients and HFD-fed mice, as well as in steatotic liver cells in vitro. APP expression did not correlate with Aβ-42 tissue levels or histopathologic inflammation. Therefore, APP expression, which plays a role in cholesterol turnover [28], may be linked to lipid homeostasis, but its expression level may not be causative for Aβ-42 tissue concentration in the livers of patients with MASLD.

APP can be metabolized via the amyloidogenic pathway, but also via the non-amyloidogenic pathway, by α-secretases such as ADAM9, ADAM10, and ADAM17, thereby reducing APP levels for further processing towards Aβ generation [2]. In samples from patients with MASH, we found an increased expression of three α-secretases, ADAM9, ADAM10, and ADAM17, with an inverse correlation with Aβ-42 levels. Previous reports point to the enhanced hepatic expression of ADAM10 [35] and ADAM17 [36] in mouse models of MASLD, most likely reflecting their involvement in MASLD progression. The activities of ADAM10 and ADAM17 in mouse livers with MASH are negatively regulated by reversion-inducing cysteine-rich proteins with Kazal motifs (RECKs), membrane-anchored glycoproteins [37], which were recently shown to be reduced in samples from patients with MASLD [38]. Additionally, BACE2 expression is enhanced in MASH tissues, particularly in those with severe fibrosis, as recently reported for the pediatric population with liver fibrosis [10]. BACE2, a homologous aspartase of BACE1, is primarily expressed in the peripheral tissues and cleaves APP within the Aβ domain near the α-secretase site, leading to diminished Aβ production [39]. In summary, liver tissue from patients with advanced MASLD revealed an increased expression of genes known to cleave APP towards the non-amyloidogenic pathway, such as ADAM9, ADAM10, ADAM17, and BACE2, which are likely involved in decreased hepatic Aβ-42 levels in MASLD.

Aβ-42 is generated through the sequential cleavage of APP by BACE1, which mediates the initial and rate-limiting processing steps, and the γ-secretase complex containing presenilin (PS) 1/2. We found a reduced BACE1 expression in fibrotic livers of patients with MASLD, which is in line with the reduced BACE1 mRNA expression in cirrhotic livers [9] and might be associated with upregulated Nrf2 (NFE2L2), known for its negative BACE1 regulation [31], in patients with MASLD [32]. PS1 expression is enhanced in MASH samples and seems to be linked to steatosis, whereas its expression is related to the histopathological steatosis grade. Of note, UCHL1 (Ubiquitin C-terminal hydrolase 1), an important player in the elimination of oxidized or misfolded proteins and known to decrease BACE1-catalyzed cleavage [40], is increasingly expressed in the tissue samples of patients with MASH and severe fibrosis, and inversely correlates with Aβ-42 levels. A reduced UCHL1 activity is related to increased BACE1 and Aβ peptide accumulation in the brain [40]; UCHL1 was found to be upregulated in hepatic stellate cells upon activation in fibrosis, as well as in samples from patients with alcoholic liver disease [41]. In summary, reduced Aβ-42 levels in liver tissue from patients with MASLD might be due to limited Aβ-42 production because the expression of its rate-limiting enzyme BACE1 is decreased and UCHL1, a negative BACE1 regulator, is enhanced.

Several proteins facilitate the transport of Aβ peptides between different compartments from the brain to the liver. Among the analyzed genes that influence hepatic Aβ transport and Aβ levels in MASLD, we found almost no change in the expression of A2M (alpha-2-macroglobulin) [33], APOA1 (apolipoprotein A1) [42], APOJ (apolipoprotein J; clusterin) [33], Deptor (DEP domain-containing mTOR-interacting protein) [43], TTR (transthyretin) [44], or LRP1 (LDL receptor-related protein 1) [33]. Apolipoprotein E (APOE) can regulate Aβ transport, clearance, and aggregation, and the expression of specific isoforms of APOE (E4 over E3 and E2) is responsible for a genetic predisposition to AD [33]. We found an increased expression of APOE in samples with higher steatosis grades, but it was lower along with more severe fibrosis, which is reflected in the higher APOE expression in livers with a low compared to a high MAS. In addition, we analyzed a variety of genes involved in Aβ degradation and that are associated with AD development, which has been described in detail recently (reviews [4,33]). In MASH samples, we found an enhanced expression of ACE, MMP2, MMP7, and MMP9, of which ACE, MMP2, and MMP7 were inversely correlated with the corresponding Aβ-42 tissue levels, indicating a regulatory role for Aβ-42 in MASLD. Elevated MMP2 mRNA expression in MASH samples has been reported previously [45] and might be explained by RECK expression (see ADAM10 and ADAM17 above), a negative regulator of MMP2 and MMP9 [37], which is reduced in samples from patients with MASLD [38]. In line with this, we found an increased ACE, MMP2, and MMP7 mRNA expression, but also reduced APEH, ECE1, MBP, and NEP in samples with an advanced fibrosis grade. Overall, an increased expression of metalloproteinases, ACE, and MMP, which are known to degrade Aβ peptides and are associated with fibrosis, might contribute to lower Aβ-42 tissue levels in MASLD.

The significance of the liver–brain axis in AD has been shown previously, reflected by MASLD-induced neurodegeneration [6,21] or cirrhosis-impaired Aβ-42 clearance [7]. In this study, we highlighted the impact of MASLD on the regulation of hepatic Aβ-42 levels, which has been shown to protect against liver fibrosis/cirrhosis [9,10]. The deposition of lipids and steatosis accompanied by oxidative stress are responsible for the regulation of genes involved in APP and Aβ metabolism, but have no implication on hepatic Aβ-42 levels. In liver samples from patients with MASH, we found reduced Aβ-42 concentrations, which were not dependent on the expression of APP or Aβ transport proteins, but most likely caused by (i) an increased degradation of APP towards the non-amyloidogenic pathway, (ii) a degraded generation of Aβ peptides along the amyloidogenic pathway, and (iii) an enhanced expression of genes removing Aβ-42. Incipient fibrosis is paralleled by reduced Aβ-42 liver tissue levels in MASLD, and low Aβ-42 may favor hepatic fibrogenesis [10], but the initial cause of Aβ decline in liver fibrosis remains to be determined.

## 4. Materials and Methods

*Study Subjects and Collection of Samples:* Liver tissue samples of patients without MASLD (n = 26), patients with simple liver steatosis (n = 30), and patients with MASH (n = 36) were examined and analyzed, as described previously [16] (for tissue characteristics, see Supplementary Tables S1 and S2). The experimental procedures were performed according to the guidelines of the charitable state-controlled foundation HTCR (Human Tissue and Cell Research, Regensburg, Germany), with written informed consent from patients. The study and the consent form were approved by the local ethical committee of the University of Regensburg (ethics statement 12-101-0048, University of Regensburg, Germany). All experiments involving human tissues and cells have been carried out in accordance with *The Code of Ethics of the World Medical Association* (Declaration of Helsinki).

*Mouse liver tissue:* Mouse liver samples were obtained from mice, as described in a previously published study [17]. Briefly, fourteen-week-old male C57BL/6 mice were fed ad libitum a control diet (ssniff^®^ EF acc. D12450B (I) mod., SD) or a high-fat diet (ssniff^®^ EF R/M, D12451, 42% of energy from fat, HFD) for 14 weeks (n = 5 per group). Procedures were approved by the University of Regensburg Laboratory Animal Committee and complied with the German Law on Animal Protection and the Institute for Laboratory Animal Research Guide for the Care and Use of Laboratory Animals, 1999. Experiments were conducted according to institutional and governmental regulations for animal use (Government of the Oberpfalz and Unterfranken, 54-2532.1-30/13, 9 December 2013).

*Cell culture and treatments:* In vitro experiments were performed, as previously described [23]. The human hepatoma cell line HepG2 was obtained from American Type Culture Collection (HB-8065, ATCC, Manassas, VA, USA) and Huh7 cells (ECACC 01042712) were obtained from the European Collection of Authenticated Cell Cultures (ECACC) (Salisbury, UK). Cells were grown at 37 °C and 5% CO_2_ in DMEM supplemented with 1% MEM (100x), 1% L-glutamine (200 mM), 1% penicillin/streptomycin (10,000 U/mL), and 10% fetal calf serum (all from Sigma-Aldrich, Deisenhofen, Germany). Cells were seeded at a density of 5 × 10^4^ cells/cm^2^ in 6-well plates and were maintained in culture for 24 h. After a period of starvation (serum-free culture medium DMEM: HepG2 for 24 h, Huh7 for 8 h), cells were treated with indicated concentrations of either PA or with a PA/OA mixture (ratio 1:2) for 24 h. Free fatty acids (OA, #01008-5G, PA #P0500-10G) were obtained from Sigma-Aldrich (Deisenhofen, Germany), were dissolved in isopropanol, and added to culture medium supplemented with 1% BSA (Roth, Karlsruhe, Germany). PHHs were isolated and maintained in culture, as described earlier [46]. 

*Real-Time quantitative PCR (SYBR Green):* The mRNA expression was investigated using real-time qPCR using SYBR Green, as described recently [9]. Total RNA was isolated from cultured cells or mouse liver tissue using the RNeasy Mini Kit (Qiagen, Hilden, Germany), and an additional on-column DNase digestion with RNase-Free DNase Set (Qiagen, Hilden, Germany) and was quantified at 260/280 nm with the Thermo Fisher Scientific Nanodrop 2000 spectrophotometer. One μg of total RNA was reverse-transcribed using the QuantiTect Reverse-Transcription System (Qiagen, Hilden, Germany) according to the manufacturer’s instructions. Real-time RT-PCR (Roche, Penzberg, Germany) was performed in triplicate using the LightCycler ^®^ 480 SYBR Green I Master (Roche), and the specificity of the PCR reactions was confirmed by sequencing the amplified DNA fragments (Geneart, Regensburg, Germany). For the quantification and analysis of the efficiency of each PCR reaction, serially diluted standard curves (at least six different dilutions for each of the genes analyzed) were calculated. The PCR reaction was evaluated using dissociation curve analysis, and expression values were normalized to the expression values of the housekeeping gene YWAHZ (tyrosine 3-monooxygenase/tryptophan 5-monooxygenase activation protein zeta) or HPRT1 (hypoxanthine guaninephosphoribosyl transferase (1)). The primers used for qRT-PCR are listed in Tables S4 and S5.

*SDS-PAGE and immunoblotting:* The total protein concentration was determined using the BCA Protein assay (Bio-Rad). Briefly, 50 μg of protein per lane was separated using 12% SDS-PAGE (Bio-Rad, Hercules, CA, USA) under reducing conditions, and proteins were transferred onto PVDF membranes (Amersham Cytiva, ThermoFisher Scientific, Darmstadt, Germany). Membranes were blocked in 5% BSA (Albumin Fraction V, Applichem, Darmstadt, Germany) in TBS-0.5%Tween (Merck, Darmstadt, Germany) for 1 h, incubated at 4 °C overnight with respective primary antibodies diluted in 5%BSA/TBS-0.5%Tween, and were developed using enhanced chemiluminescence reagent (Thermo Fisher Scientific, Darmstadt, Germany). The following antibodies were used: anti-APP (#SAB3500274; 1:4000) was obtained from Sigma-Aldrich (Taufkirchen, Germany), anti-neprilysin (#ab79423; 1:1000) from Abcam (Cambridge, UK), anti-BACE1 (#PA1-757; 1:500) and anti-PS1 (#PA5-20376; 1:750) from Thermo Fisher Scientific (Waltham, MA, USA), and anti-β-actin (#4970; 1:2000) from Cell Signaling (Danvers, MA, USA). Secondary goat horseradish peroxidase-conjugated antibodies (anti-rabbit #P0448; 1:10,000) were obtained from Dako (Hamburg, Germany). A VersaDocTM 4000 MP imaging system (Bio-Rad, Hercules, CA, USA) was used for imaging and densitometric analyses. The data were normalized to the respective densitometric values of β-actin as a loading control.

*Taqman quantitative Real time PCR (qRT-PCR):* Total RNA was isolated from a larger cohort of human liver tissues (see Supplementary Table S1) using the RNeasy kit, including on-column genomic DNA digestion with the RNase-free DNase Set (Qiagen, Hilden, Germany). For quantitative RT-PCR analysis, we used the Fluidigm BioMark high-throughput quantitative (q) PCR chip platform (Fluidigm Corporation, San Francisco, CA, USA) with pre-designed gene expression assays from Thermo Fisher (Supplementary Table S3), according to the manufacturer’s instructions. The data were analyzed using the ΔΔCt method, and the expression values were normalized to the expression levels of three housekeeping genes (*HPRT1, GUSB,* and *TBP*).

*Quantification of Aβ-42 Peptides in Liver homogenates:* In human (for characteristics, see Supplementary Table S2) and mouse livers, Aβ-42 peptides were detected using the V-Plex^®^ Kit (Mesoscale, Rockville, MD, USA) using the antibody (4G8) recognizing human and rodent Aβ-42. Mouse livers with their respective controls were analyzed using the Luminex assay using the MILLIPLEX MAP Mouse Amyloid Beta Magnetic Bead Kit (MABMAG-83K, Merck).

*Statistical analysis:* mRNA expression results of patient samples were evaluated for normality distribution using a Shapiro–Wilk test. Data were presented as box plots displaying median values, lower and upper quartiles, and the range of the values. Statistical differences between two groups were analyzed using a two-tailed Mann–Whitney U Test or Student’s unpaired t-test (in vitro), and between several groups (data from human samples) using a Kruskal–Wallis test with post hoc Bonferroni correction where appropriate. Values of *p* < 0.05 were considered significant (SPSS Statistics 26.0 program, IBM, Leibniz Rechenzentrum, München, Germany). Each experiment was performed at least in triplicate, and results were expressed as means ± SD (standard deviation) or SEM (standard error of the mean), as indicated.

## Data Availability

The original contributions presented in this study are included in the article/supplementary material; further inquiries can be directed to the corresponding author.

## Supplementary Materials

The following supporting information can be downloaded at: https://www.mdpi.com/article/10.3390/ijms25168768/s1. References [46,47,48,49,50,51,52,53,54,55,56,57,58,59] are cited in the supplementary materials.
