# Supplementary material for: Hepatic Amyloid Beta-42-Metabolizing Proteins in Liver Steatosis and Metabolic Dysfunction-Associated Steatohepatitis
Source: Int J Mol Sci. 2024 Aug 12;25(16):8768. doi: 10.3390/ijms25168768 (PMC11354580; doi:10.3390/ijms25168768)
Supplement: Supplementary file 1 [file ijms-25-08768-s001.zip › ijms-3121045-supplementary.pdf]

# Hepatic amyloid beta-42 metabolizing proteins in liver steatosis and metabolic dysfunction-associated steatohepatitis

**Simon Gross[1], Lusine Danielyan[2], Christa Buechler[3], Marion Kubitza[1], Kathrin Klein[4], Matthias Schwab[2,4,5], Michael Melter[1], Thomas S. Weiss[1, 6, *]**

**Supplementary Materials**

**Supplementary Figure**

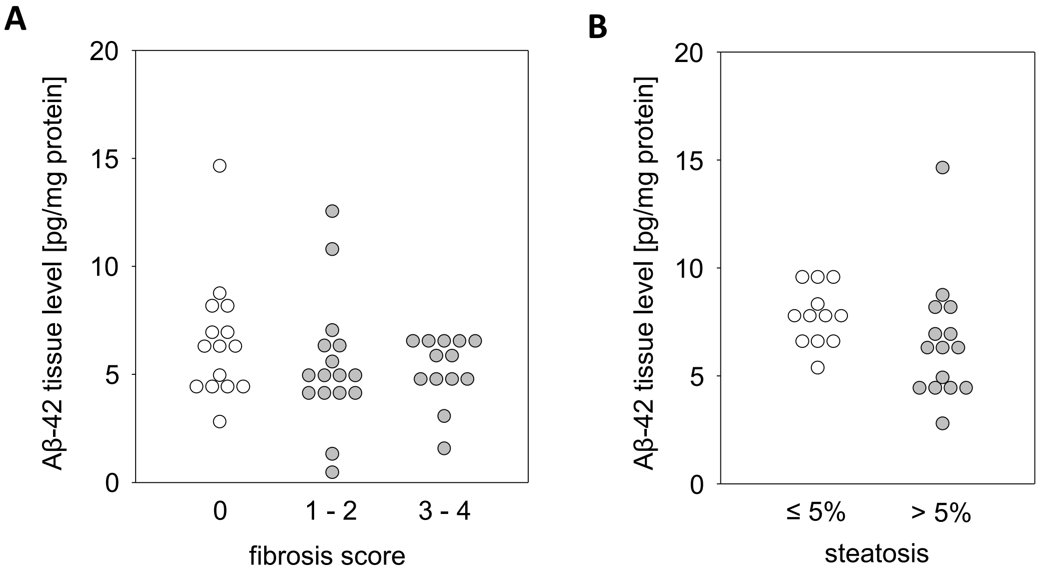


Figure S1: A) Aβ-42 liver tissue concentrations in samples from patients with MAS > 1 (n=44) plotted regarding fibrosis score: 0 (no fibrosis; n=15; 6.52 ± 2.83), 1-2 (mild to moderate fibrosis; n= 16; 5.42 ± 2.98) and 3-4 (severe fibrosis to cirrhosis, n=13; 5.25 ± 1.55). B) Aβ-42 tissue concentrations in liver samples without fibrosis (n=27) and with steatosis: ≤ 5% steatotic cells (n=12; 7.78 ± 1.35) and ≥ 5% steatotic cells (n=15; 6.52 ± 2.83). * $p < 0,05$, ** $p < 0,01$, were considered as statistically different.

**Tables**

Table S1: Characteristics of liver samples used for gene expression (qRT-PCR) analysis. Age, body mass index (BMI), steatosis, inflammation- and fibrosis scores, ALT, type 2 diabetes (T2D), hypertension, hypercholesterinemia of the cohort studied. Data are shown as median (range of values).

| | *Control* | *steatosis* | *MASH* |
|---|---|---|---|
| Patients (female/male) | 26 (15 / 11) | 30 (18 / 12) | 36 (15 / 21) |
| Age [years] | 59 (20-72) | 61.5 (24-77) | 67 (41-81) |
| BMI [kg/m$^2$] | 24.9 (18-42) | 27.0 (21-46) | 28.4 (22-58) |
| Steatosis [a] | 0 | 2 (1-3) | 2 (1-3) |
| Inflammation [b] | 0 | 0 (0-1) | 2 (1-3) |
| Fibrosis [c] | 0 | 0 (0-4) | 2 (0-3) |
| Ballooning [d] | 0 | 1 (1-1) | 2 (1-2) |
| MASH activity score [e] | 0 (0) | 3 (1-4) | 6 (5-8) |
| ALT [U/L] | 18.5 (8-50) | 29.5 (14-984)b | 32.0 (10-411) |
| T2D (no/yes) | 26 / 0 | 26 / 4 | 25 / 11 |
| Hypertension (no/yes) | 19 / 7 | 20 / 10 | 19 / 17 |
| Hypercholesterinemia (no/yes) | 26 / 0 | 28 / 2 | 29 / 7 |

Table S2: Characteristics of liver samples used for tissue Aβ-42 analysis. Age, body mass index (BMI), steatosis, inflammation- and fibrosis scores, ALT, type 2 diabetes (T2D), hypertension, hypercholesterinemia of the cohort studied for gene expression. Data are shown as median (range of values).

| | *Control* | *steatosis* | *MASH* |
|---|---|---|---|
| Patients (female/male) | 12 (7 / 5) | 21 (11 / 10) | 23 (10 / 13) |
| Age [years] | 57 (20-71) | 64 (35-77) | 67 (42-82) |
| BMI [kg/m$^2$] | 24.2 (18-31) | 27.0 (22-46) | 29.0 (22-58) |
| Steatosis [a] | 0 | 2 (1-2) | 3 (1-3) |
| Inflammation [b] | 0 | 0 (0-1) | 2 (1-3 |
| Fibrosis [c] | 0 | 0 (0-4) | 2 (0-3) |
| Ballooning [d] | 0 | 1 (0-1) | 2 (1-2) |
| MASH activity score [e] | 0 (0) | 3 (1-4) | 6 (5-8) |
| ALT [U/L] | 18.5 (10-50) | 29.0 (14-984) | 31.5 (10-77) |
| T2D (no/yes) | 12 / 0 | 17 / 4 | 13 / 10 |
| Hypertension (no/yes) | 8 / 4 | 12 / 9 | 11 / 12 |
| Hypercholesterinemia (no/yes) | 12 / 0 | 19/ 2 | 18 / 5 |

Human liver tissues for mRNA expression analysis were histologically examined for patients without MASLD, patients with simple liver steatosis and patients with MASH as described earlier [1]. Anamnesis excluded alcohol intake, drugs and viral infections as cause for MASLD. Surgery was done because of hepatic metastases of extrahepatic tumors (56 patients), focal nodular hyperplasia of the liver (5 patients), adenoma (6 patients), cholangiocarcinoma (11 patients), hepatocellular carcinoma (10 patients) and other diseases (4 patients) and only non-affected tissue was used. In particular, liver samples without pathological findings (without MASLD, normal liver) were obtained from resected tissue of patients undergoing surgery because of hepatic metastases of extrahepatic tumors (20 patients), focal nodular hyperplasia of the liver (2 patients), cholangiocarcinoma (3 patients) and other diseases (3 patients).

[a] Steatosis was scored as <5% steatosis (0), 5% to 33% steatosis (1), >33% to 66% steatosis (2) and >66% steatosis (3).

[b] Inflammation was scored as no foci / 20 × field (0), <2 foci / 20 × field (1), 2–4 foci / 20 × field (2) and >4 foci / 20 × field (3).

[c] Fibrosis was defined as no fibrosis (0), zone 3 perisinusoidal/ pericellular fibrosis; focally or extensively present (1), zone 3 perisinusoidal/ pericellular fibrosis with focal or extensive

periportal fibrosis (2), zone 3 perisinusoidal/ pericellular fibrosis and portal fibrosis with focal or extensive bridging fibrosis (3) and cirrhosis (4).

[d] Ballooning was scored by the number of enlarged hepatocytes as none / field (0), few ballooned hepatocytes / field (1), many ballooned hepatocytes / field (2).

[e] MASH activity was calculated and assigned to MASH activity score (MAS, from 0 to 8) as following: 0, normal; 1-2, no MASH; 3-4, no MASH; 5-6, mild MASH and 7-8, severe MASH. Patients with a MAS score 1 - 4 were assigned as steatosis and those with ≥ 5 as suffering from MASH, respectively.

Table S3. List of genes and assay numbers (Thermo Fisher) used for Fluidigm qRT-PCR.

| *Gene* | *Gene Name* | *Assay ID* |
|---|---|---|
| APP | amyloid beta precursor protein | Hs00169098_m1 |
| *Non-amyloidogenic pathway, degradation of APP* | | |
| ADAM9 | ADAM metallopeptidase domain 9 | Hs00177638_m1 |
| ADAM10 | ADAM metallopeptidase domain 10 | Hs00153853_m1 |
| ADAM17 | ADAM metallopeptidase domain 17 | Hs01041915_m1 |
| BACE2 | beta-site APP-cleaving enzyme 2 | Hs00273238_m1 |
| HMGCS2 | 3-hydroxy-3-methylglutaryl-CoA synthase 2 | Hs00985427_m1 |
| *Amyloidogenic pathway* | | |
| BACE1 | beta-secretase 1 | Hs01121195_m1 |
| PS1 / PSEN1 | presenilin 1 | Hs00997789_m1 |
| PS2 / PSEN2 | presenilin 2 | Hs01577197_m1 |
| UCHL1 | ubiquitin C-terminal hydrolase L1 | Hs00985157_m1 |
| *Degradation of Aβ-42* | | |
| APEH | acylaminoacyl-peptide hydrolase | Hs00155756_m1 |
| ACE | angiotensin I converting enzyme | Hs00174179_m1 |
| CTSB | cathepsin B | Hs00947433_m1 |
| CST3 | cystatin C | Hs00969174_m1 |
| ECE1 | endothelin converting enzyme 1 | Hs01043735_m1 |
| IDE | insulin degrading enzyme | Hs00610452_m1 |
| MBP | myelin basic protein | Hs00921945_m1 |
| MMP2 | matrix metallopeptidase 2 | Hs01548727_m1 |
| MMP7 | matrix metallopeptidase 7 | Hs01042796_m1 |
| MMP9 | matrix metallopeptidase 9 | Hs00957562_m1 |
| NEP / MME | Nepreselin / membrane metallo-endopeptidase | Hs00153510_m1 |
| *Transport / binding of Aβ-42* | | |
| A2M | alpha-2-macroglobulin | Hs00929971_m1 |
| APOA1 | apolipoprotein A1 | Hs00163641_m1 |
| APOE | apolipoprotein E | Hs00171168_m1 |
| APOJ / CLU | Apolipoprotein J / clusterin | Hs00156548_m1 |
| DEPTOR | DEP domain containing MTOR-interacting protein | Hs00961900_m1 |
| LRP1 | LDL receptor related protein 1 | Hs00233856_m1 |
| TTR | transthyretin | Hs00174914_m1 |
| *Substrates of γ-secretase* | | |
| NOTCH1 | notch 1 | Hs01062014_m1 |
| NOTCH3 | notch 3 | Hs00166432_m1 |
| *Housekeeping genes* | | |
| GUSB | glucuronidase beta | Hs99999908_m1 |
| HPRT1 | hypoxanthine phosphoribosyltransferase 1 | Hs02800695_m1 |
| TBP | TATA-box binding protein | Hs00427620_m1 |

Table S4. Human primer for RT-qPCR (SYBR Green).

| *Human* | *Accession Nr.* | *Primer sequence (5' – 3')* | *Reference* |
|---|---|---|---|
| APP | NM_000484.3 | Fwd. GCC AAA GAG ACA TGC AGT GA | [2] |
| | | Rev. CCA GAC ATC CGA GTC ATC CT | |
| ADAM9 | NM_003816.3 | Fwd. ACT GTG AAA ATG GCT GGG CT | [3] |
| | | Rev. ACC AGA AGT CCG TCC CTC AAT G | |
| ADAM10 | NM_001110 | Fwd. CTG CCC AGC ATC TGA CCC TAA | [4] |
| | | Rev. TTG CCA TCA GAA CTG GCA CAC | |
| ADAM17 | NM_003183 | Fwd. GGT TCC TTT CGT GCT GGC GC | [5] |
| | | Rev. AAG CTT CTC GAG TCT CTG GTG GG | |
| BACE1 | NM_004048 | Fwd. AAGTTCATTACCTCCCTATCAGT | [6] |
| | | Rev. AGGCCCTCCTTGTATTTCC | |
| NEP / MME | NM_000902.5 | Fwd. CCCAGTGCATGGTGTATCAG | [2] |
| | | Rev. TGGCCTATAGGTTCCACACC | |
| PS1 / PSEN1 | NM_000021.4 | Fwd. CCT CAA CAA TGG TGT GGT TG | [7] |
| | | Rev. TTG TGA CTC CCT TTC TGT GCT | |
| HPRT1 | NM_000194 | Fwd. TGA CAC TGG CAA AAC AAT GCA | [8] |
| | | Rev. CCT TTT CAC CAG CAA GCT | |

Table S5. Mouse primer for RT-qPCR (SYBR Green).

| *Mouse* | *Accession Nr.* | *Primer sequence (5' – 3')* | *Reference* |
|---|---|---|---|
| APP | NM_001198823 | Fwd. CCG TTG CCT AGT TGG TGA GT | [9] |
| | | Rev. GCT CTT CTC GCT GCA TGT C | |
| ADAM9 | NM_007404 | Fwd. GGA TAT GGA GGA AGC GTG GA | [10] |
| | | Rev. GCA ACA AGG GGG ACG ATT AG | |
| ADAM10 | NM_007399 | Fwd. AGC AAC ATC TGG GGA CAA AC | [10] |
| | | Rev. TGG CCA GAT TCA ACA AAA CA | |
| ADAM17 | NM_001277266.1 | Fwd. GTA CGT CGA TGC AGA GCA AA | [10] |
| | | Rev. GAA ATC CCA AAA TCG CTC AA | |
| BACE1 | NM_011792 | Fwd. GCT TGC ACC TGT AGG ACA CA | [11] |
| | | Rev. CTA AAG GAT GCTG GGC AGA G | |
| NEP / MME | NM_001289462 | Fwd. GTA AGC AGC CTC AGC CGA AAC | [12] |
| | | Rev. CCA CAT AAA GCC TCC CCA CAG | |
| PS1 / PSEN1 | NM_001362271 | Fwd. AAG TAC CTC CCC GAA TGG AC | [13] |
| | | Rev. TCA GCC ATA TTC ACC AAC CA | |
| NOTCH1 | NM_008714 | Fwd. ACA GTG CAA CCC CCT GTA TG | [14] |
| | | Rev. TCT AGG CCA TCC CAC TCA CA | |
| NOTCH3 | NM_008716 | Fwd. TTG TCT GGA TGG AAG CCC ATG T | [15] |
| | | Rev. ACT GAA CTC TGG CAA ACG CCT | |
| YWHAZ | NM_011740 | Fwd. CGC TAA TAA TGC AGT TAC TGA GAG A | [1] |
| | | Rev. TTG GAA GGC CGG TTA ATT TT | |

YWHAZ, tyrosine 3-monooxygenase/tryptophan 5-monooxygenase activation protein zeta.

Table S6. Correlation of Aβ-42 tissue level with mRNA expression of genes involved in APP metabolism in liver tissue samples with MASH (MAS ≥ 5, n = 23).

| | Aβ-42 tissue level | | | Aβ-42 tissue level | |
|---|---|---|---|---|---|
| | *r* | *p-value* | | *r* | *p-value* |
| APP | -0.068 | 0.757 | | | |
| *Non-amyloidogenic pathway. degradation of APP* | | | *Degradation of Aβ-42* | | |
| | | | APEH | 0.222 | 0.309 |
| ADAM9 | -0.569 | 0.005 ** | ACE | -0.418 | 0.047 * |
| ADAM10 | -0.477 | 0.021 * | CTSB | 0.256 | 0.239 |
| ADAM17 | -0.418 | 0.047 * | CST3 | -0.211 | 0.334 |
| BACE2 | -0.271 | 0.211 | ECE1 | 0.219 | 0.314 |
| HMGCS2 | 0.423 | 0.049 | IDE | -0.007 | 0.974 |
| *Amyloidogenic pathway* | | | MBP | -0.387 | 0.068 |
| BACE1 | 0.320 | 0.137 | MMP2 | -0.477 | 0.021 * |
| UCHL1 | -0.439 | 0.036 * | MMP7 | -0.561 | 0.005 ** |
| PS1 / PSEN1 | -0.109 | 0.619 | MMP9 | -0.050 | 0.822 |
| PS2 / PSEN2 | -0.207 | 0.356 | NEP / MME | 0.534 | 0.009 |
| *Transport / binding of Aβ-42* | | | *Substrates of γ-secretase* | | |
| A2M | 0.311 | 0.148 | NOTCH1 | -0.191 | 0.382 |
| APOA1 | 0.241 | 0.281 | NOTCH3 | -0.409 | 0.053 |
| APOE | 0.259 | 0.232 | | | |
| APOJ / CLU | 0.343 | 0.109 | | | |
| DEPTOR | -0.087 | 0.693 | | | |
| LRP1 | 0.340 | 0.112 | | | |
| TTR | 0.472 | 0.026 * | | | |

r = Pearson correlation coefficient, * $p < 0.05$, ** $p < 0.01$.

Table S7. Correlation of liver tissue inflammation status with mRNA expression of genes involved in APP metabolism in liver tissue samples with steatosis or MASH (MAS ≥ 1, n = 66).

| | Inflammation | | | Inflammation | |
|---|---|---|---|---|---|
| | r | p-value | | r | p-value |
| APP | 0.109 | 0.382 | | | |
| *Non-amyloidogenic pathway. degradation of APP* | | | *Degradation of Aβ-42* | | |
| ADAM9 | 0.426 | 0.000 ** | APEH | -0.276 | 0.025 * |
| ADAM10 | 0.269 | 0.029 * | ACE | 0.253 | 0.040 * |
| ADAM17 | 0.283 | 0.022 * | CTSB | -0.171 | 0.170 |
| BACE2 | 0.231 | 0.062 | CST3 | 0.243 | 0.049 * |
| HMGCS2 | -0.404 | 0.001 ** | ECE1 | -0.016 | 0.898 |
| *Amyloidogenic pathway* | | | IDE | -0.042 | 0.738 |
| BACE1 | -0.290 | 0.018 * | MBP | 0.143 | 0.253 |
| UCHL1 | 0.297 | 0.016 * | MMP2 | 0.437 | 0.000 ** |
| PS1 / PSEN1 | 0.153 | 0.221 | MMP7 | 0.345 | 0.005 ** |
| PS2 / PSEN2 | 0.178 | 0.156 | MMP9 | 0.269 | 0.029 * |
| *Transport / binding of Aβ-42* | | | NEP / MME | -0.321 | 0.009 ** |
| A2M | -0.016 | 0.896 | *Substrates of γ-secretase* | | |
| APOA1 | -0.185 | 0.139 | NOTCH1 | 0.200 | 0.107 |
| APOE | -0.318 | 0.009 ** | NOTCH3 | 0.335 | 0.006 ** |
| APOJ / CLU | -0.296 | 0.016 * | | | |
| DEPTOR | -0.033 | 0.791 | | | |
| LRP1 | -0.210 | 0.091 | | | |
| TTR | -0.236 | 0.061 | | | |

r = Pearson correlation coefficient, * $p < 0.05$, ** $p < 0.01$.

**References**


1. Weiss TS, Lupke M, Ibrahim S, Buechler C, Lorenz J, Ruemmele P, et al. Attenuated lipotoxicity and apoptosis is linked to exogenous and endogenous augmenter of liver regeneration by different pathways. PLoS One. 2017;12(9):e0184282. Epub 2017/09/07. doi: 10.1371/journal.pone.0184282.
2. Huang H, Bihaqi SW, Cui L, Zawia NH. In vitro Pb exposure disturbs the balance between Abeta production and elimination: the role of AbetaPP and neprilysin. Neurotoxicology. 2011;32(3):300-6. doi: 10.1016/j.neuro.2011.02.001.
3. Zhu LJ, Zhao YY, Yu L, He XJ, Wang YJ, Jiang P, et al. Overexpression of ADAM9 decreases radiosensitivity of hepatocellular carcinoma cell by activating autophagy. Bioengineered. 2021;12(1)(1):5516-28.
4. Pan B, Huo T, Cao M, Jing L, Luo X, Qu Z, et al. ADAM10 promotes the proliferation of ligamentum flavum cells by activating the PI3K/AKT pathway. International journal of molecular medicine. 2021;47(2):688-98.
5. Arcidiacono P, Webb CM, Brooke MA, Zhou H, Delaney PJ, Ng K-E, et al. p63 is a key regulator of iRHOM2 signalling in the keratinocyte stress response. Nature communications. 2018;9(1):1021.
6. Wang T, Shi F, Jin Y, Jiang W, Shen D, Xiao S. Abnormal Changes of Brain Cortical Anatomy and the Association with Plasma MicroRNA107 Level in Amnestic Mild Cognitive Impairment. Front Aging Neurosci. 2016;8:112. Epub 2016/06/01. doi: 10.3389/fnagi.2016.00112.
7. Buniatian GH, Weiskirchen R, Weiss TS, Schwinghammer U, Fritz M, Seferyan T, et al. Antifibrotic Effects of Amyloid-Beta and Its Loss in Cirrhotic Liver. Cells. 2020;9(2). Epub 2020/02/25. doi: 10.3390/cells9020452.
8. Fischer M, Skowron M, Berthold F. Reliable transcript quantification by real-time reverse transcriptase-polymerase chain reaction in primary neuroblastoma using normalization to averaged expression levels of the control genes HPRT1 and SDHA. J Mol Diagn. 2005;7(1):89-96.
9. Augustin S, Rimbach G, Augustin K, Schliebs R, Wolffram S, Cermak R. Effect of a short- and long-term treatment with Ginkgo biloba extract on amyloid precursor protein levels in a transgenic mouse model relevant to Alzheimer's disease. Archives of biochemistry and biophysics. 2009;481(2):177-82.
10. Kim DY, Lee M, Kim EJ. Involvement of Klotho, TNF-alpha and ADAMs in radiation-induced senescence of renal epithelial cells. Molecular Medicine Reports. 2021;23(1)(1):-.
11. Wen Y, Yu WH, Maloney B, Bailey J, Ma J, Marie I, et al. Transcriptional regulation of beta-secretase by p25/cdk5 leads to enhanced amyloidogenic processing. Neuron. 2008;57(5):680-90.
12. Kalinin S, Gavrilyuk V, Polak PE, Vasser R, Zhao J, Heneka MT, et al. Noradrenaline deficiency in brain increases beta-amyloid plaque burden in an animal model of Alzheimer's disease. Neurobiol Aging. 2007;28(8):1206-14.
13. Hajdu M, Luttun A, Pelacho B, Burns TC, Chase L, Gutierrez-Perez M, et al. Transcriptional characterization of the Notch signaling pathway in rodent multipotent adult progenitor cells. Pathology oncology research : POR. 2007;13(4):302-10.
14. Robinson SC, Klobucar K, Pierre CC, Ansari A, Zhenilo S, Prokhortchouk E, et al. Kaiso differentially regulates components of the Notch signaling pathway in intestinal cells. Cell Commun Signal. 2017;15(1):24. Epub 2017/06/24. doi: 10.1186/s12964-017-0178-x.
15. Baeten JT, Lilly B. Differential Regulation of NOTCH2 and NOTCH3 Contribute to Their Unique Functions in Vascular Smooth Muscle Cells. J Biol Chem. 2015;290(26):16226-37. Epub 2015/05/10. doi: 10.1074/jbc.M115.655548.
